# Turmeric extract and its active compound, curcumin, protect against chronic CCl_4_-induced liver damage by enhancing antioxidation

**DOI:** 10.1186/s12906-016-1307-6

**Published:** 2016-08-26

**Authors:** Hwa-Young Lee, Seung-Wook Kim, Geum-Hwa Lee, Min-Kyung Choi, Han-Wool Jung, Young-Jun Kim, Ho-Jeong Kwon, Han-Jung Chae

**Affiliations:** 1 Department of Pharmacology and New Drug Development Institute, Chonbuk National University Medical School, Jeonju, Chonbuk 561-180 Republic of Korea; 2 CS1 Center, Ottogi Research Center, Ottogi Corporation, Kyeonggi-do, Republic of Korea; 3 Food Safety Center, Ottogi Corp, 49 Heungan-daero 395 beon-gil, Dongan-gu, Anyang-si, Gyeonggi-do 14060 South Korea; 4 Chemical Genomics National Research Laboratory, Department of Biotechnology, Translational Research Center for Protein Function Control, College of Life Science and Biotechnology, Yonsei University, Seoul, 120-752 Republic of Korea

**Keywords:** Curcumin, Carbon tetrachloride, Oxidative stress, Glutathione, Lipid peroxidase

## Abstract

**Background:**

Curcumin, a major active component of turmeric, has previously been reported to alleviate liver damage. Here, we investigated the mechanism by which turmeric and curcumin protect the liver against carbon tetrachloride (CCl_4_)-induced injury in rats. We hypothesized that turmeric extract and curcumin protect the liver from CCl_4_-induced liver injury by reducing oxidative stress, inhibiting lipid peroxidation, and increasing glutathione peroxidase activation.

**Methods:**

Chronic hepatic stress was induced by a single intraperitoneal injection of CCl_4_ (0.1 ml/kg body weight) into rats. Turmeric extracts and curcumin were administered once a day for 4 weeks at three dose levels (100, 200, and 300 mg/kg/day). We performed ALT and AST also measured of total lipid, triglyceride, cholesterol levels, and lipid peroxidation.

**Result:**

We found that turmeric extract and curcumin significantly protect against liver injury by decreasing the activities of serum aspartate aminotransferase and alanine aminotransferase and by improving the hepatic glutathione content, leading to a reduced level of lipid peroxidase.

**Conclusions:**

Our data suggest that turmeric extract and curcumin protect the liver from chronic CCl_4_-induced injury in rats by suppressing hepatic oxidative stress. Therefore, turmeric extract and curcumin are potential therapeutic antioxidant agents for the treatment of hepatic disease.

**Electronic supplementary material:**

The online version of this article (doi:10.1186/s12906-016-1307-6) contains supplementary material, which is available to authorized users.

## Background

Liver diseases constitute a major global problem. Carbon tetrachloride (CCl_4_) is a well-known hepatotoxin that is widely used to induce acute or chronic toxic liver injury in a large range of laboratory animals [1, 2]. Chronic hepatotoxicity induces oxidative damage, necrosis, and inflammation in the liver [3]. The best-characterized model of xenobiotic-induced free radical-mediated liver disease is CCl_4_-induced liver damage in rats [4]. A recent study reported that CCl_4_ induces hepatic damage via reactive oxygen species (ROS) [5]. Elevated ROS production, together with inhibition of the antioxidant system, can generate a state of oxidative stress that leads to cell damage [6].

*Curcuma longa Linn* (CLL, turmeric), an herb widely farmed in Asia, is a primary constituent of a traditional Chinese medicine [7] that has been used effectively for centuries to treat liver diseases in China. Curcumin, best known as a yellow pigment in CLL, has been found to have antioxidant, anti-inflammatory, anti-hepatotoxic, and anti-cancer properties [8–10]. Recently, curcumin has been indicated as a potential treatment for liver damage through mediation of various signaling pathways. It decreased the expression of pro-inflammatory mediators through down regulation of toll-like receptor 4 (TLR4) and TLR2 expression in CCl_4_-induced rat model of fibrogenesis [11] and Curcumin could also remarkably attenuate the severity of CCl_4_-induced liver damage through suppression of TGF-β1/Smad signaling pathway and CTGF expression [12]. Another studies demonstrated that curcumin inhibited activation of HSC in vitro by reducing cell proliferation, inducing apoptosis and suppressing ECM gene expression [13].

Here we investigated the role of turmeric and its active compound, curcumin, in liver protection in a rat model of CCl_4_-induced damage. We hypothesized that turmeric extract and curcumin protect the liver from CCl_4_-induced damage by reducing oxidative stress, decreasing lipid peroxidation, and suppressing glutathione activation. We demonstrate that the hepatoprotective mechanisms of turmeric extract and curcumin involve the regulation of oxidative stress.

## Methods

### Materials

CCl_4_ and Curcumin were purchased from Sigma-Aldrich (St. Louis, MO, USA). Malondialdehyde (MDA) and glutathione (GSH) detection kits were obtained from BioVision (Mountain View, CA, USA). Aspartate aminotransferase (AST), alanine aminotransferase (ALT), total cholesterol, triglyceride, HDL cholesterol, and LDL-cholesterol detection kits were obtained from Asan Pharmaceutical Company (Seoul, Korea).

### Animal Treatment and Care

Seventy male Sprague-Dawley rats weighing 250–270 g were obtained from central lab. animal Inc. (Seoul, Korea) and divided into 7 groups. Rats were intraperitoneally (i.p.) injected with a mixture of CCl_4_ (0.1 mL/100 g body weight) and olive oil [1:1(v/v)] every other day for 4 weeks. Rats were orally administered turmeric at doses of 100, 200, and 300 mg/kg body weight. Curcumin was given once daily at a dose of 200 mg/kg. All rats were fed a chow diet and kept at 22–23 °C under a 12 h dark/light cycle. The control animals were handled similarly, including i.p. injection with the same volume of olive oil and oral administration of the same volume of PBS. After the last CCl_4_ injection, the rats were anesthetized with diethyl ether (Sigma) and sacrificed. All animal procedures in this study were performed in accordance with the regulations described in the Care and Use of Laboratory Animals guide of Chonbuk National University. All procedures were also approved by the Institutional Animal Care and Use Committee of Chonbuk National University for the animal center (IACUC protocol CBU 150608-25).

### Sample collection

Liver and blood samples were collected from all sacrificed animals. Whole blood was immediately placed on ice in a centrifuge tube for 30 min and spun by centrifugation at 7,168 × g for 10 min. Serum was transferred to 1.5 mL tubes and stored at −75 °C. All harvested liver tissue samples were immediately stored at -75 °C.

### Histologic analysis

Liver samples were fixed in 10 % formalin and embedded in paraffin. Liver sections were incubated for 10 min in 0.5 % thiosemicarbazide, stained with 0.1 % Sirius red F3B in saturated picric acid for 1 h, and washed with acetic acid (0.5 %). Sections were visualized using a Nikon Eclipse E600 microscope (Kawasaki, Kanagawa, Japan) at 40 × magnification, and relative areas of fibrosis (% positive areas for Sirius red staining) were quantified by histomorphometry using a computerized image analysis system (AnalySIS, Soft Imaging System, Munster, Germany). Hepatic steatosis was assessed by Oil Red O staining. Briefly, liver cryosections were fixed for 10 min in 60 % isopropanol, followed by staining with 0.3 % Oil Red O in 60 % isopropanol for 30 min and washing with 60 % isopropanol. Sections were counterstained with Gill’s hematoxylin, washed with 4 % acetic acid, and mounted in an aqueous solution. Stained sections were quantified by histomorphometry.

### Blood biochemical marker assays

Serum ALT and AST activities were measured by a colorimetric procedure using commercially available detection kits.

### Measurement of total lipid, triglyceride, and cholesterol levels

For lipid determination, liver homogenates were extracted according to the modified Bligh and Dyer procedure [14]. Briefly, samples were homogenized with chloroform-methanol-water (8:4:3), shaken at 37 °C for 1 h, and spun by centrifugation at 1,100 × *g* for 10 min. The bottom layer was collected for hepatic lipid analysis. Triglyceride, total cholesterol, and total lipid contents were measured using kits from Asan Pharmaceutical Company in accordance with the manufacturer’s instructions.

### Analyses of lipid peroxidation

Lipid peroxidation was assessed in liver tissue using a lipid hydroperoxide assay kit purchased from Cayman Chemicals (Ann Arbor, MI, USA). In this assay, lipid hydroperoxide was extracted from the samples into chloroform using the extraction buffer provided by the manufacturer. The chromogenic reaction was carried out at room temperature for 5 min and the absorbance of each well was read at 500 nm using a 96 well plate spectrometer (SpectraMax 190). 13-Hydroperoxy-octadecadienoic acid was used as the standard. The cellular levels of lipid hydroperoxide were calculated as described by the manufacturer.

### Measurement of glutathione

The levels of hepatic glutathione (GSH) and oxidized GSH (GSSG) were determined using a commercially available kit (BioVision). Briefly, tissue was homogenized and sonicated in 0.5 ml of ice cold buffer [50 mM MES (pH 7.0) with 1 mM EDTA] and then spun by centrifugation at 10,000 × *g* for 15 min at 4 °C. The supernatant was collected and deproteinized using the reagent supplied in the kit. Next, 50 μL of sample were mixed with 150 μl of assay cocktail in each well and incubated in the dark on a plate shaker. After 30 min, the absorbance of each well was measured at 404 nm. The concentration of total GSH was calculated according to the equation in the manufacturer’s protocol.

### Sirius red collagen staining

Liver tissue was fixed with 10 % formalin and embedded in paraffin. Thin sections were deparaffinized and stained with picro-Sirius red for 60 min at room temperature. Sections on slides were dehydrated sequentially in 100 % ethanol and xylene, and then mounted in Permount (Thermo Fisher Scientific, Waltham, MA, USA). Representative views of liver sections are shown.

### Statistical analysis

Results are presented as the mean ± SEM. The MicroCal Origin software (Northampton, MA, USA) was used for all statistical calculations. Differences were tested for significance using one-way analysis of variance (ANOVA) with Duncan’s multiple range test. Statistical significance was set at *p* < 0.05.

## Results

### Validation of the HPLC method

#### Linearity

The slopes, y-intercepts, and correlation coefficients (r^2^) obtained from regression analysis were as follows: The calibration curves were linear in the tested concentration ranges. The regression equations were *y* = 47.53*x* – 1.02 (*r*^*2*^ = 0.9999), for bisdemethoxycurcumin (BDMC); *y* = 44.92*x* – 14.62 (*r*^*2*^ = 0.9991), for demethoxycurcumin (DMC); and *y* = 45.52*x* – 67.42 (*r*^*2*^ = 0.9992), for curcumin, respectively. The correlation coefficients were all greater than 0.999, indicating high degrees of correlation and good linearity of the method.

#### Precision and accuracy

The intra-day and inter-day precision and accuracy for determination of curcuminoids are given in Table 1. The % RSD values for intra-day precision were 0.13, 0.10, and 0.13 for curcumin, DMC, and BDMC, respectively, and those for inter-day precision were 0.30, 0.48, and 0.59, respectively. The low values of % RSD (<0.59 %) reflect the high precision of the method. The percentage recoveries for intra-day accuracy were 95.84 ± 0.12, 98.15 ± 0.09, and 101.08 ± 0.13, respectively, and those for inter-day accuracy were 95.78 ± 0.29, 97.96 ± 0.47, and 101.74 ± 0.60, respectively. All percentage recoveries were within 95.78 – 101.083 %, indicating the good accuracy of the method.

**Table 1:** The intra-day and inter-day precision and accuracy for determination of curcuminoids

	Present (g/100 g)	Added (g/100 g)	Found (g/100 g)	Accuracy (%)	Precision (RSD%)
Intra-day					
Bisdemethoxycurcumin	0.095	0.096	0.097 ± 0.0001	101.08	0.13
Demethoxycurcumin	0.247	0.262	0.257 ± 0.0003	98.15	0.10
Curcumin	0.993	1.066	1.022 ± 0.002	95.84	0.13
Inter-day					
Bisdemethoxycurcumin	0.095	0.096	0.098 ± 0.001	101.74	0.59
Demethoxycurcumin	0.247	0.262	0.257 ± 0.001	97.96	0.48
Curcumin	0.993	1.066	1.021 ± 0.003	95.78	0.30

### Analysis of compounds in turmeric extract

HPLC analysis was used to identify the two major compounds in turmeric extract as curcumin (901.63 ± 5.37 mg/100 g), BDMC (108.28 ± 2.89 mg/100 g), and DMC (234.85 ± 1.85 mg/100 g) curcuminoids (1244.76 ± 3.86 mg/100 g) (Table 2). The representative HPLC chromatogram is presented in Additional file 1: Figure S1.

**Table 2:** Analysis of turmeric extract

Component	mg/100 g
Curcumin	901.6 ± 5.4
Curcuminoids	1244.8 ± 3.9
BDMC	108.3 ± 2.9
DMC	234.9 ± 1.9

### Turmeric extract and its active compound, curcumin, regulate body weight during chronic CCl_4_ exposure

After 4 weeks, the total body weight of each rat was measured. The average weight gain of rats in the control group was normal. In contrast, rats in the chronic CCl_4_ exposure group exhibited significantly increased weight gain compared with rats in the control group, whereas administration of turmeric extract significantly inhibited this increase (Table 3). Administration of turmeric extract at 300 mg/kg lessened body weight gain to a greater extent than that at 100 or 200 mg/kg. Moreover, administration of 200 mg/kg curcumin also significantly inhibited body weight gain. Daily food intake was monitored in all groups. The average daily food consumption in each group is shown in Table 3; importantly, no significant differences were observed between any of the groups regarding food intake. These findings suggest that both turmeric extract and curcumin attenuate CCl_4_-induced weight gain.

**Table 3:** Effects of turmeric extract and curcumin on food intake and body weight

Group	Food intake (g/day)	Body weight (g)
Control	22.1 ± 3.1	335 ± 11.2
Curcumin	23.2 ± 3.3	335 ± 10.5
300 mg/kg turmeric extract	22.0 ± 2.3	351 ± 12.2
CCl_4_	22.3 ± 3.2	401 ± 20.4
CCl_4_ + curcumin	19.9 ± 3.2	376.0 ± 18.5
CCl_4_ + 100 mg/kg turmeric extract	24.6 ± 2.0	400.4 ± 19.5
CCl_4_ + 200 mg/kg turmeric extract	23.1 ± 1.3	381.1 ± 20.3*
CCl_4_ + 300 mg/kg turmeric extract	21.2 ± 2.1	362.5 ± 20.2*

*p < 0.05 vs. the CCl_4_ group

### Both turmeric extract and curcumin regulate CCl_4_-induced lipid accumulation

The serum levels of triglyceride, total cholesterol, LDL-C, and HDL-C were also measured (Table 4). Compared with control mice, mice in the CCl_4_ group showed decreased TG, total cholesterol, and LDL-C levels. However, the levels of HDL-C were not different between the two groups. Compared with mice in the CCl_4_ group, mice treated with turmeric extract and mice treated with curcumin exhibited significantly increased TG, total cholesterol, and LDL-C levels.

**Table 4:** Serum concentrations of triglyceride, total cholesterol, LDL-C, and HDL-C as determined by quantitative lipid assays

Group	Serum levels (mg/dl)			
	TG	Total cholesterol	LDL-cholesterol	HDL-cholesterol
Control	82.4 ± 2.2	136.7 ± 12.8	56.6 ± 6.3	45.4 ± 6.4
Curcumin	89.4 ± 1.2	142.7 ± 6.3	60.2 ± 3.1	45.6 ± 1.6
300 mg/kg turmeric extract	83.7 ± 2.3	141.9 ± 1.5	63.2 ± 2.0	46.5 ± 3.3
CCl_4_	51.5 ± 7.1	94.3 ± 1.7	44.1 ± 4.0	44.5 ± 2.5
CCl_4_ + curcumin	77.0 ± 1.6	111.6 ± 8.3	52.2 ± 6.0	45.0 ± 9.4
CCl_4_ + 100 mg/kg turmeric extract	75.1 ± 1.0	95.2 ± 9.4	46.6 ± 7.1	43.8 ± 1.8
CCl_4_ + 200 mg/kg turmeric extract	78.1 ± 2.5*	101.3 ± 3.6*	49.6 ± 3.1*	45.4 ± 1.5
CCl_4_ + 300 mg/kg turmeric extract	83.0 ± 1.5*	112.3 ± 9.1*	51.5 ± 8.0*	44.8 ± 2.6

*p < 0.05 vs. the CCl_4_ group

### Both turmeric extract and curcumin protect against CCl_4_-induced liver damage

**Fig. 1:**
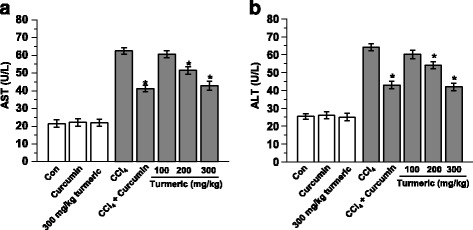
Turmeric extract and curcumin reduce AST and ALT levels in CCl_4_-induced hepatic failure. Rats were intraperitoneally injected with CCl_4_ (0.1 mL/100 g body weight) every other day for 4 weeks. Turmeric extract (100, 200, and 300 mg/kg) and curcumin (200 mg/kg) were given once daily. Liver and blood samples were collected from all sacrificed animals. Serum levels of AST (**a**) and ALT (**b**) were determined. ^#^ *p* < 0.05 vs. the CCl_4_ group

**Fig. 2:**
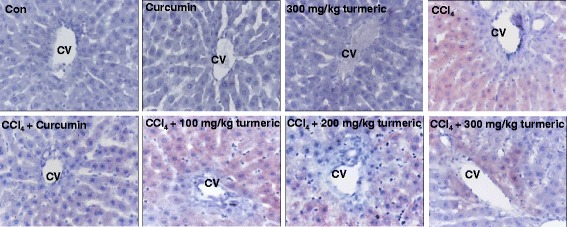
Turmeric extract and curcumin protect the liver from CCl_4_-induced damage and lipid accumulation. Rats were intraperitoneally injected with CCl_4_ (0.1 mL/100 g body weight) every other day for 4 weeks. Turmeric extract (100, 200, and 300 mg/kg) and curcumin (200 mg/kg) were given once daily. Liver and blood samples were collected from all sacrificed animals. Liver tissue was fixed and stained with Oil Red O

To evaluate the protective effects of turmeric extract and curcumin against CCl_4_-induced lipid accumulation and liver damage, we performed histologic analyses of the livers of treated mice. CCl_4_ administration significantly increased the levels of the liver damage biomarkers aspartate transaminase (AST) and alanine transaminase (ALT) (Fig. 1). However, treatment with turmeric extract (300 mg/kg) or curcumin (200 mg/kg) significantly decreased the AST and ALT levels, indicating reduced liver damage compared with the CCl_4_ group. Morphologic analysis showed that CCl_4_ administration stimulated steatosis, as indicated by the appearance of lipid droplets. However, treatment with turmeric extract (300 mg/kg) or curcumin (200 mg/kg) resulted in decreased steatosis compared with the CCl_4_ group (Fig. 2).

### Turmeric extract and curcumin regulate CCl_4_-induced lipid peroxidation and antioxidant activity

**Fig. 3:**
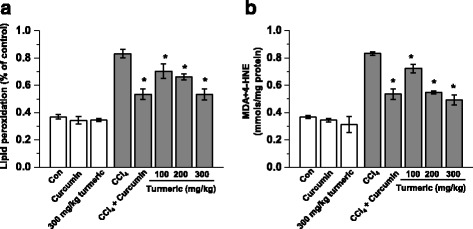
Turmeric extract and curcumin protect the liver from CCl_4_-induced lipid peroxidation. Rats were intraperitoneally (i.p.) injected with CCl_4_ (0.1 mL/100 g body weight) every other day for 4 weeks. Turmeric extract (100, 200, and 300 mg/kg) and curcumin (200 mg/kg) were given once daily. After liver samples were collected from all sacrificed animals, the levels of lipid peroxidation (**a**) and MDA + 4-HNE (**b**) were measured. ^#^ *p* < 0.05 vs. the CCl_4_ group

**Fig. 4:**
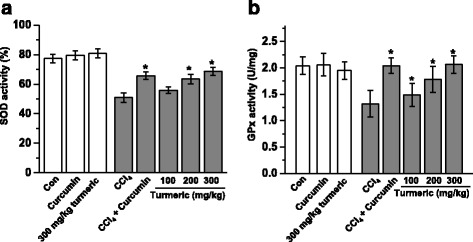
Turmeric extract and curcumin protect the liver from CCl_4_-induced oxidative stress. Rats were intraperitoneally injected with CCl_4_ (0.1 mL/100 g body weight) every other day for 4 weeks. Turmeric extract (100, 200, and 300 mg/kg) and curcumin (200 mg/kg) were given once daily. After liver samples were collected from all sacrificed animals, the levels of SOD (**a**) and GPx (**b**) were measured. ^#^ *p* < 0.05 vs. the CCl_4_ group

We next measured hepatic lipid peroxide levels (Fig. 3a). The levels of the hepatic lipid peroxides malondialdehyde (MDA) and 4-hydroxynonenal (4-HNE) were markedly increased in the CCl_4_ group compared with the control group. However, both turmeric extract and curcumin alleviated the increased levels of the lipid peroxides MDA and 4-HNE (Fig. 3b). We also quantified hepatic antioxidant activities (Fig. 4). Hepatic superoxide dismutase (SOD) and glutathione peroxidase (GPx) activities were markedly reduced in the CCl_4_ group compared with the control group; however, these effects were reversed by both turmeric extract and curcumin.

### Turmeric extract and curcumin regulate CCl_4_-induced oxidative stress in the liver

**Fig. 5:**
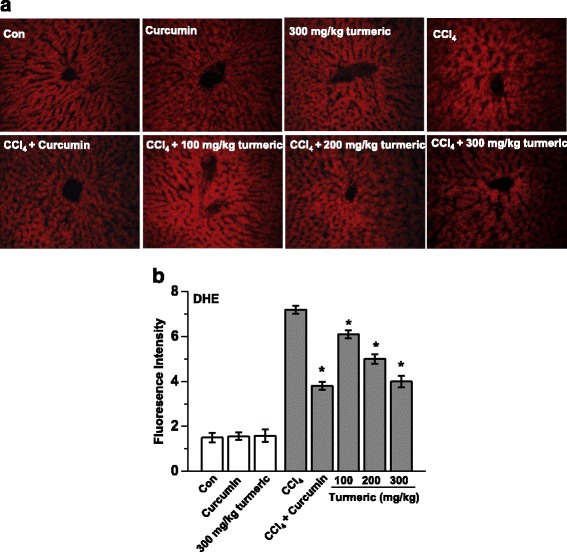
Turmeric extract and curcumin protect the liver from CCl_4_-induced ROS production. Rats were intraperitoneally injected with CCl_4_ (0.1 mL/100 g body weight) every other day for 4 weeks. Turmeric extract (100, 200, and 300 mg/kg) and curcumin (200 mg/kg) were given once daily. **a** Liver tissue was isolated and loaded with 5 μM dihydroethidium. Fluorescence images were acquired. **b** Quantitative fluorescence density data. ^#^ *p* < 0.05 vs. the CCl_4_ group

**Fig. 6:**
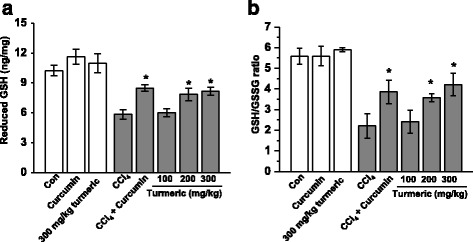
Turmeric extract and curcumin protect the liver from CCl_4_-induced oxidative stress. Rats were intraperitoneally injected with CCl_4_ (0.1 mL/100 g body weight) every other day for 4 weeks. Turmeric extract (100, 200, and 300 mg/kg) and curcumin (200 mg/kg) were given once daily. **a** Livers were isolated and the levels of reduced glutathione (GSH) (**a**) and the GSH/GSSG ratios (**b**) were determined. ^#^ *p* < 0.05 vs. the CCl_4_ group

Oxidative stress resulting from CCl_4_ treatment contributes to liver injury and stimulates hepatic fibrosis. The effects of turmeric and curcumin on CCl_4_-induced oxidative stress in the liver are shown in Fig. 5. In these experiments, red fluorescence from dihydroethidium indicates an increased ROS content in the liver. Chronic CCl_4_ exposure resulted in increased fluorescence, whereas much lower fluorescence was observed in the livers of rats treated with turmeric extract or 200 mg/kg curcumin. Moreover, these effects were dose-dependent. Glutathione (GSH) is the major low-molecular weight thiol and the most critical nonenzyme antioxidant in vitro [15]. GSH protects cells against oxidative stress-induced cellular damage by removing hydrogen peroxide (H_2_O_2_) and inhibiting lipid peroxidation [16]. The GSH/GSSG ratio is considered to be a sensitive indicator of oxidative stress [17, 18]. To evaluate the impact of turmeric extract and curcumin on oxidative stress in vivo, the levels of hepatic GSH and the ratios of reduced GSH to GSSG were determined. As shown in Fig. 6, CCl_4_ treatment markedly decreased the level of total GSH and the GSH/GSSG ratio. However, oral administration of turmeric and of curcumin completely prevented these CCl_4_-induced effects, resulting in levels of total hepatic GSH and hepatic GSH/GSS ratios similar to those of control rats. These results suggest that both turmeric extract and curcumin can protect the liver against CCl_4_-induced damage by attenuating oxidative stress.

## Discussion

In the present study, we showed that chronic CCl_4_-induced liver injury, defined as increased levels of serum markers of hepatic damage and abnormal liver morphology, was inhibited in the presence of turmeric extract and its active compound, curcumin. Moreover, the hepatic GSH/GSSG ratio was restored in the turmeric extract and its active compound, curcumin-treated group, indicating that this extract alleviates oxidative stress.

Both turmeric extract and its active compound, curcumin, remarkably decreased CCl_4_-induced liver damage. Curcumin is a polyphenolic compound found in the dietary spice *Curcuma longa Linn*. Due to its wide range of biological and biochemical activities, the therapeutic effects of curcumin are being investigated in various disease models, including hepatic failure [19–21]. The main components of turmeric extract include curcumin and other curcuminoids (Table 2). Because curcumin is poorly absorbed following oral administration and is not hydrosoluble, the majority of the ingested curcumin is excreted intact after passing through the digestive tract. We thus selected a high dose of curcumin, 200 mg/kg, during experimental design. Other studies used a similar dose of curcumin [22–24]. In these studies, curcumin was reported to be metabolized at the intestinal mucosa and liver, with curcumin plasma levels detectable only when it was administered at a gram-based high dose; ≥ 1 g [25, 26]. In this study, orally administered curcumin was expected to be metabolized to a greater degree in the CCl4-exposed condition. Based on other references [22–24], we selected a high dose of curcumin, 200 mg/kg. Throughout this study, curcumin was used as a functional component control. The CCl_4_-induced experimental model of liver damage is very useful for the study of hepatotoxic effects [27] since CCl_4_ consistently produces liver damage in many species, including nonhuman primates [28]. In the liver, CCl_4_ metabolism stimulates lipid peroxidation and upregulates ROS production [29], which is important because peroxidated lipids and ROS cause hepatocyte necrosis, induce inflammation, and further stimulate the progression of hepatic fibrosis. In the progression of chronic hepatic failure, lipid accumulation is also frequently observed and is ultimately linked to the fibrosis pattern. However, elevated serum ALT and AST levels, clear markers of liver injury, were not observed in the turmeric extract-treated group (Fig. 1a, b). Similarly, curcumin also inhibited the increases in aminotransferase levels. Other studies have also demonstrated that curcumin administration can reverse the elevated serum AST and ALT levels in models of hepatotoxicity [30]. The current study showed that both turmeric extract and curcumin alleviate CCl_4_-induced upregulation of serum ALT and AST, indicating liver protection from CCl_4_-induced toxicity. In addition, turmeric extract and curcumin attenuated CCl_4_-mediated hepatic lipid accumulation (Fig. 2). The levels of serum TG, total cholesterol, and LDL were significantly decreased in the CCL_4_ group, whereas they were restored in the turmeric extract-treated group (Table 4). The hepatic lipid profiles (Table 5) were consistent with the serum profiles and the Oil Red O staining data (Fig. 2). Moreover, curcumin exerted a protective effect similar to that of the highest dose of the extract. This study shows that turmeric extract and curcumin protect the liver from CCl_4_-induced hepatic lipid accumulation. However, hepatic fibrosis was not observed in this model (data not shown). We conclude that more severe stress conditions and a longer time frame than this 4-weeks system are required to investigate aspects of fibrosis.

**Table 5:** Hepatic concentrations of triglyceride, total cholesterol, LDL-C, and HDL-C as determined by quantitative lipid assays

Group	Liver levels (mg/dl)			
	TG	Total cholesterol	LDL-cholesterol	HDL-cholesterol
Control	60.6 ± 0.1	70.6 ± 5.2	50.5 ± 4.3	44.3 ± 2.5
Curcumin	68.1 ± 0.1	68.9 ± 1.3	48.5 ± 8.5	47.1 ± 4.6
300 mg/kg turmeric extract	61.3 ± 0.2	71.3 ± 1.6	45.8 ± 5.5	45.6 ± 1.3
CCl_4_	92.7 ± 0.2	99.6 ± 1.5	84.6 ± 7.6	44.9 ± 7.8
CCl_4_ + curcumin	78.9 ± 0.2*	75.4 ± 1.5*	62.5 ± 5.6*	46.2 ± 1.5
CCl_4_ + 100 mg/kg turmeric extract	87.1 ± 0.1	92.7 ± 2.0	82.2 ± 5.5	45.6 ± 5.6
CCl_4_ + 200 mg/kg turmeric extract	77.3 ± 0.1*	88.3 ± 4.6*	75.5 ± 7.6*	45.0 ± 2.8
CCl_4_ + 300 mg/kg turmeric extract	68.3 ± 0.1*	78.1 ± 1.4*	61.5 ± 7.6*	46.5 ± 1.9

*p < 0.05 vs. the CCl_4_ group

Oxidative stress is closely associated with hepatic lipid accumulation and liver failure [31]. Lipid peroxidation is markedly suppressed in the livers of rats treated with antioxidants such as flavonoids and vitamins [32]. We hypothesized that turmeric extract and curcumin protect the liver against CCl_4_-induced injury and hepatic lipid accumulation by decreasing oxidative stress. In support of our hypothesis, treatment with turmeric extract and treatment with curcumin both reduced oxidative stress, as demonstrated by the reductions in the CCl_4_-mediated increases in lipid peroxidation, malondialdehyde (MDA), and 4-hydroxynonenal (4-HNE) levels (Fig. 3a, b). Our results show that turmeric extract and curcumin not only increase the level of total hepatic GSH, but also markedly improve the GSH/GSSG ratio (Fig. 6). Although many factors have been implicated in CCl_4_-induced liver damage, oxidative stress and ROS production are thought to be of primary importance. Oxidative stress is defined as an imbalance between ROS production and removal [33]. ROS, which are generated as products of oxidative metabolism, frequently damage cellular macromolecules such as DNA and lipids. In the present study, the accumulation of ROS in the liver after CCl_4_ treatment was ameliorated by treatment with turmeric extract and treatment with curcumin (Fig. 5a, b). These results strongly suggest that turmeric extract and curcumin protect against hepatic functional disturbances, including hepatic dysmetabolism, through ROS regulation.

## Conclusions

This study suggests that turmeric extract and curcumin are both highly effective in preventing chronic CCl_4_-induced liver damage and provides new insights into the potential pharmacologic targets of curcumin in the prevention of liver disease.

## Additional file

Additional file 1: Figure S1. Chromatograms of blank (A), standard (B), turmeric extract (C). (PDF 164 kb)

## Abbreviations

4-HNE: 4-hydroxynonenal
AST: Aspartate transaminase
CCl_4_: Carbon tetrachloride
CLL: *Curcuma longa L. turmeric*
GPx: Glutathione peroxidase
MDA: Malondialdehyde
ROS: Reactive oxygen species
SOD: Superoxide dismutase
