# Supplementary material for: Turmeric extract and its active compound, curcumin, protect against chronic CCl4-induced liver damage by enhancing antioxidation
Source: BMC Complement Altern Med. 2016 Aug 26;16(1):316. doi: 10.1186/s12906-016-1307-6 (PMC5000414; doi:10.1186/s12906-016-1307-6)

Turmeric extract and its active compound, curcumin, protect against chronic CCl_4-induced liver damage by enhancing antioxidation

Hwa-Young Lee[1, #], Seung-Wook Kim[2, #], Geum-Hwa Lee[1], Min-Kyung Choi[1], Han-Wool Jung[1], Ho-Jeong Kwon[3], Han-Jung Chae[1, *]

[1]Department of Pharmacology and New Drug Development Institute, Chonbuk National University Medical School, Jeonju, Chonbuk 561-180, Republic of Korea; [2]CS1 Center, Ottogi Research Center, Ottogi Corporation, Kyeonggi-do, Republic of Korea; [3]Chemical Genomics National Research Laboratory, Department of Biotechnology, Translational Research Center for Protein Function Control, College of Life Science and Biotechnology, Yonsei University, Seoul 120-752, Republic of Korea

[#]These authors contributed equally to this work.

Supplementary Materials and Method

Reagents and Chemicals

The chemicals used were all analytical grade. Pure reference standards of curcumin, demethoxycurcumin, and bisdemethoxycurcumin were supplied by Chromadex Co. (Irvine, CA).

Preparation of Standard Solutions.

Individual standard solutions were prepared by dissolving 5 mg of each compound in 10 mL of methanol to obtain a final concentration of 500 mg/L. The working standard solutions were prepared at concentrations of 0.2, 1.0, 5, 10, 25, 50, and 100 mg/L by serially diluting the curcuminoid solutions in methanol to fit the calibration curves and determine the linearity of the responses.

Quantitation of curcumin and curcuminoids using HPLC-DAD

The HPLC analysis of individual curcuminoids including bisdemethoxycurcumin, demethoxycurcumin and curcumin was performed according to a previous report (Kim *et al.*, 2013). Briefly, 100 mg of turmeric extract powder was weighed, dissolved in 30 mL methanol in a 250 mL round-bottomed flask, and refluxed for 60 min. The extract was cooled, transferred to a 100 mL volumetric flask, and filtered through a 0.45 μm filter. A 1200 HPLC series (Agilent Technologies, Palo Alto, CA, USA) consisted of a degasser, pump, autosampler, column oven and photodiode array detector. A Capcell Pak C18 UG120 column (4.6 mm i.d. × 250 mm, 5 μm Shiseido, Inc., Tokyo, Japan) was used as the stationary phase at a column temperature of 25 °C. The mobile phase consisted of 1.0% acetic acid in Milli-Q water (solvent A) and 100% acetonitrile (solvent B). The flow rate was 1.0 mL/min and a linear gradient elution from 48% solvent A and 52% solvent B to 50% solvent B in 10 min. An aliquot (5 μL) of this extract was injected into the HPLC system. Individual curcuminoids were identified by comparing their retention time with those of corresponding standards. The determination was performed three times, and the results were recorded as mg/100 g.

Linearity

Linearity of the detector response was verified with bisdemethoxycurcumin, demethoxycurcumin , and curcumin standard solutions over the range of 0.2−100 mg/L. Calibration curves were prepared each experiment day, and the concentration of the analytes in the samples was calculated.

Precision and Accuracy

The intraday precision of the HPLC−DAD method was tested six times/day with a quality control sample (fortified turmeric solution), which was a standard solution of bisdemethoxycurcumin, demethoxycurcumin , and curcumin. For interday precision, three measurements/day on three different days were conducted. The precision of the method was expressed as the relative standard deviation (RSD) for the repeated measurements. The accuracy of the method was calculated as the relative difference
between the determined and nominal concentrations of the analyzed samples.

Supplementary Figure 1. Chromatograms of blank (A), standard (B), turmeric extract (C).

Supplementary Figure 1

A

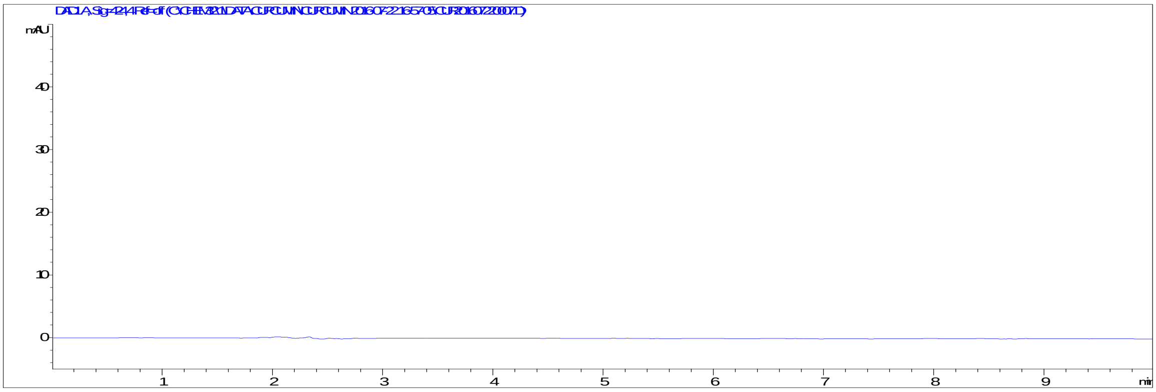

B

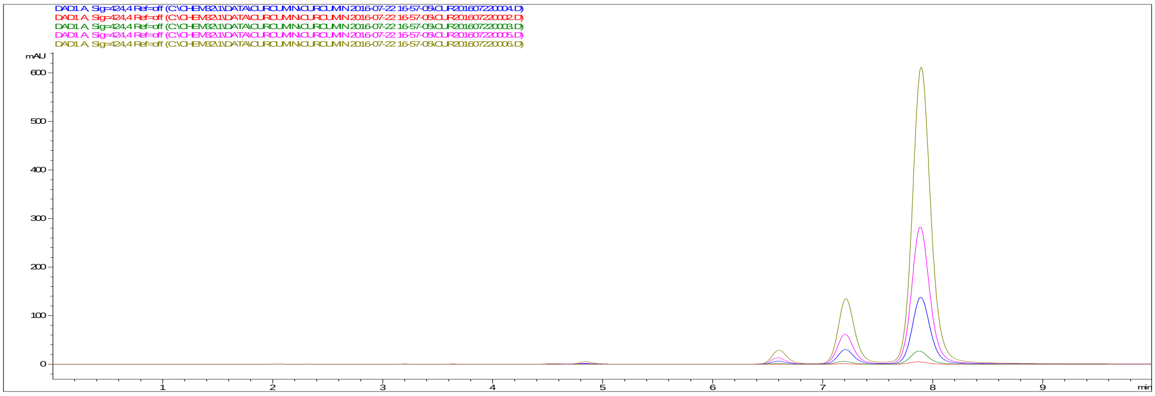

C

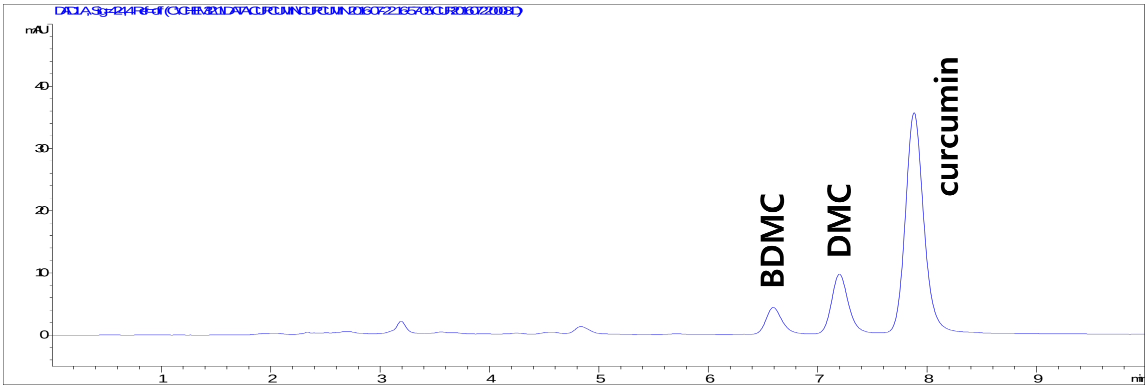

Supplement: Additional file 1: Figure S1. — Chromatograms of blank (A), standard (B), turmeric extract (C). (PDF 164 kb) [file 12906_2016_1307_MOESM1_ESM.pdf]
